# Cell Therapy Ameliorates Cortical Circuit Dysfunction in an Alzheimer's Disease Model

**DOI:** 10.1002/alz70855_098890

**Published:** 2025-12-23

**Authors:** Shinya Yokomizo, Megi Maci, April Stafford, Morgan R. Miller, Stephen J. Perle, Shusaku Takahashi, Heather Brown‐Harding, Linda Liang, Alex Lovely, Moustafa Algamal, Rebecca L. Gillani, Theodore J. Zwang, Douglas Richardson, Janice R. Naegele, Daniel Vogt, Ksenia V. Kastanenka

**Affiliations:** ^1^ Massachusetts General Hospital, Charlestown, MA, USA; ^2^ Michigan State University, Grand Rapids, MI, USA; ^3^ Harvard University, Cambridge, MA, USA; ^4^ Harvard Medical School, Boston, MA, USA; ^5^ Massachusetts General Hospital, Boston, MA, USA; ^6^ Wesleyan University, Middletown, CT, USA

## Abstract

**Background:**

The prevalence of Alzheimer's disease is rising. While disease modifying therapies have been made available to patients, these therapeutics pose serious health risks. Therefore, finding new treatment strategies is necessary. Alzheimer's patients and those with mild cognitive impairment frequently report sleep disturbances which are linked to disease progression. Our earlier work revealed deficits in NREM sleep and impairments in slow oscillations, a brain rhythm prevalent during NREM sleep, in 6 months old APP/PS1 mice. Dysfunction of endogenous GABAergic interneurons was responsible for these sleep deficits. We thus hypothesized that transplantation of healthy interneurons would lead to survival and incorporation of donor cells into existing host circuitry. Furthermore, cell therapy would rescue slow oscillations in APP/PS1 mice.

**Method:**

Medial ganglionic eminence (MGE) precursors from VGAT‐Venus, VGAT‐ChR2‐EYFP, or Thy1‐GCaMP6f mouse embryos were harvested and injected into cortices of APP/PS1 hosts. We examined the persistence and migration of transplanted cells after tissue clearing using 3D light‐sheet microscopy. Maturation of the donor cells was assessed with interneuron markers. To assess synaptic integration, we stained with synaptic markers and imaged the samples using super resolution microscopy. The activity of the donor interneurons was determined using multiphoton calcium imaging. Finally, we determined the effects of cell transplantation on slow oscillations using voltage‐sensitive dye imaging, in absence and presence of optogenetic boost.

**Result:**

Transplanted donor cells survived, migrated within the cortices of APP/PS1 hosts. Donor cells matured into interneurons that expressed LHX6, SST and PV, but not the CGE marker PROX1. The dendritic‐like structures of donor cells exhibited inhibitory synapse clusters expressing vGAT, Bassoon, and Gephyrin. Multiphoton microscopy revealed calcium transients within the donor interneurons suggesting successful integration into host neural circuits. Importantly, cell transplantation effectively rescued slow oscillations in APP/PS1 hosts.

**Conclusion:**

These results suggest that a single administration of MGE precursors to anterior cortex rescued a sleep‐dependent brain rhythm. Thus, stem cell therapy is a viable approach for reversing functional deficits in cortical circuits of APP/PS1 mice. This work highlights that stem cell therapy holds promise for Alzheimer's patients to rescue sleep impairments and potentially slow disease progression.

